# Female-headed households contending with AIDS-related hardship in rural South Africa

**DOI:** 10.1016/j.healthplace.2010.12.017

**Published:** 2011-03

**Authors:** Enid Schatz, Sangeetha Madhavan, Jill Williams

**Affiliations:** a Departments of Occupational Therapy and Women's & Gender Studies, University of Missouri, 420 Lewis Hall, Columbia, MO 65203, USA; b MRC/Wits Rural Public Health and Health Transitions Research Unit (Agincourt), School of Public Health, Faculty of Health Sciences, University of the Witwatersrand, Johannesburg, 7 York Road, Parktown 2193, South Africa; c African American Studies Department, Maryland Population Research Center, 2169 LeFrak Hall University of Maryland, College Park, MD 20742, USA; d African Population Studies Research and Training Program, Institute of Behavioral Science, University of Colorado, Boulder 483 UCB, Boulder, CO 80309, USA

**Keywords:** Africa, South Africa, Female-headship, HIV/AIDS, Households, Gender

## Abstract

Mainstream research and the popular media often equate female-headship with household vulnerability, crisis, and disorganization. Epidemic levels of HIV/AIDS in some parts of sub-Saharan Africa compound this portrait of hopelessness. In South Africa, the impact of HIV/AIDS on households depends on race, class, and place. As female-headed households increase in number, we need to better understand how female-heads in poor rural areas contend with AIDS related challenges. We analyze qualitative interviews with 16 female heads and the members of their households in a rural community to examine the response to AIDS-related illness, death, or caring for orphaned children. Our analysis examines female-heads' financial and social resources and how these resources buffer against hardship in households affected by AIDS. We find considerable heterogeneity among rural female-headed households and their access to resources to combat AIDS-related hardship. Our findings have important policy implications both in terms of identifying individual and household vulnerabilities as well as leveraging the potential for resilience for female-heads in rural South African communities.

## 1 Introduction

In developing countries, there has been a steady increase in the number of female-headed households (Bongaarts, 2001). Even though reasons for this trend are geographically and historically determined, much of the concern surrounding female-headed households assumes a link between growing numbers and the feminization of poverty. This discourse suggests that female-headed households are the “poorest of the poor” and in need for various forms of intervention (Chant, 2003; Momsen, 2002). Female-headed households are often reported as being more dependent heavy, and having low socio-economic status (Chant, 2007; Posel, 2001). Most literature on female-headship in Africa focuses on children's wellbeing and households' economic wellbeing (e.g. Buvinic, 1997; Desai, 1992; Onyango et al., 1994). However, these studies fail to reveal the heterogeneity of female-headed households. In fact, most poverty analyses do not disaggregate groups of women; rather, they assume that all female-headed households are vulnerable and poor.

Feminist scholars, on the other hand, have deconstructed female-headed households bringing attention to variation and complexity (Chant, 1997, 2003; Datta and McIlwaine, 2000; Fuwa, 2000; Varley, 1996). Three important critiques of this literature remain, however. The first is the tendency to treat households as unified entities without recognition of intra-household tensions or power dynamics (Clark, 1984; Folbre, 1986). The second is the treatment of households as bounded units to the exclusion of investigating the extended family and inter-household connections in which households are embedded (Guyer, 1981). The third is the need to situate female-headed households in appropriate historical and geographic contexts (Goebel et al., 2010). These shortcomings mask variation in vulnerability and risk within and across contexts, which may, in turn, affect our understanding of the relationship between poverty, vulnerability, and female headship.

In South Africa women head nearly half of all households (Department of Health, Medical Research Council, OrCMacro, 2007). The primary reasons for female-headship have traditionally included male labor migration and non-marriage (Posel, 2001). Although female headship in this context is connected to “historical patterns of patriarchy and apartheid” that are unique to South Africa, female-headship increasingly is connected to “contemporary macro-economic conditions” (Goebel et al., 2010, p. 578) and premature death brought on by HIV/AIDS (Gilbert et al., 2010). South Africa's adult HIV-prevalence is 10.9% (Shisana et al., 2009), which is comparable to the rates in other southern African countries. By focusing on female-headed households in a rural South African community at a time of increase in AIDS-mortality, we can show the heterogeneity of female headed households and how social environments and networks support or undermine the coping capacity of those impacted by the disease.

Using qualitative interviews with female-heads and household members in Agincourt, South Africa, we examine how inter- and intra-household relationships affect the management of AIDS-related disruptions brought on by the experience of recent AIDS-related death, the care of children orphaned by AIDS, or the care of a person living with AIDS. Our analysis gives primacy to the lived experience of female heads, highlights the dynamic nature of managing hardships related to HIV/AIDS, and provides insights for policy development.

## 2 Race, space, HIV/AIDS, and female-headship in rural South Africa

Apartheid comprised of a range of policies and practices that created gendered, racial, and spatial segregation in South Africa. Apartheid policies included forcible relocation of African populations into rural areas (called homelands) and severe restrictions on mobility (Coovadia et al., 2009). While African men were recruited to work in urban areas, their families were not allowed to join them, creating large streams of male temporary labor migration from rural homelands to urban townships, which left African women as heads of rural households (Collinson, 2009; Posel et al., 2006). Under apartheid both urban townships and rural homelands were left underdeveloped, reinforcing a racialized spatial economy whereby the rural Black population provided the needed labor to support an urban, predominantly White population (Coovadia et al., 2009). This circulation of labor has blurred the line between rural and urban experiences in South Africa (Collinson, 2009; Coovadia et al., 2009).

**Fig. 1:**
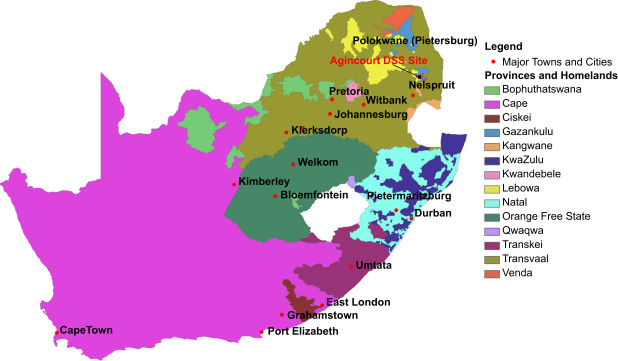
Agincourt Unit Research Site in pre-1994 South Africa.

Even though apartheid was dismantled in 1990, the lack of rural development during apartheid continues to affect former homelands through endemic poverty, underdeveloped employment opportunities, and poor access to health and welfare services (Coovadia et al., 2009), resulting in continued streams of circular migration (Allison and Harpham, 2002; Campbell et al., 2008; Feldacker et al., 2010). Fig. 1 shows the geographic separation between the former homelands and the major urban centers. The rural research site for this study (Agincourt) is situated in the former homeland of Gazankulu, approximately 500 km northeast of Johannesburg.

According to Hunter et al. (2007), the settlement pattern of the former Gazankulu area is typical of northeastern South Africa with “distinct villages surrounded by fields used for grazing and harvesting of natural resources” such as fuelwood and wild foods (p. 167). The region is semi-arid (annual rainfall 550–700 mm) but heavily populated (∼170 persons per sq. km), making water shortage a serious problem (Hunter et al., 2007). Household garden plots supplement food supply but are too small to support subsistence farming and household food shortages are common (Hunter et al., 2007; Leroy et al., 2001).

The history and geography of rural northeast South Africa, then, interact to create substantial household social and economic insecurity. Additionally, the racialized spatial economy has also proved an important factor in the spread of HIV and the availability of treatment for AIDS, further destabilizing rural households in the former homelands. AIDS has struck southern Africa harder than other parts of sub-Saharan Africa (see http://www.aidsinafrica.net/map.php), and within South Africa, HIV-prevalence varies greatly by province due in part to labor migration (Gilbert and Walker, 2009).

A growing literature details how HIV/AIDS affects families and households (Dayton and Ainsworth, 2004; Feldacker et al., 2010; Hosegood et al., 2007; Schatz and Ogunmefun, 2007; Williams and Tumwekwase, 2001). Most studies suggest that AIDS drastically alters household organization and challenges households' capacity to cope with the disease. These drastic effects are partly due to the considerable economic and emotional shocks to households and broader social networks brought on by AIDS. African households function as the primary locus of care for the sick and for the children orphaned by AIDS. There is little doubt that these households face enormous strain from costs related to illness, funerals, and care for orphaned children (Campbell et al., 2008; Ford and Hosegood, 2005; Madhavan et al., in press; Steinberg et al., 2002). Although some research has examined the burdens AIDS places on women in general (Greig and Koopman, 2003; Gupta and Weiss, 1993; Schatz and Ogunmefun, 2007), few studies have investigated how the disease may impact rural households headed by women (FAO (Food and Agriculture Organization of the United Nations), 2003; Goebel et al., 2010).

When a male head dies of an AIDS-related illness, his wife or mother may become the head. Women—mothers, grandmothers, wives, daughters, and aunts—are the most likely to become the primary caregivers for the sick and for orphaned children, whether within their own homes or beyond (Dayton and Ainsworth, 2004; Steinberg et al., 2002). In this role, women heads are likely to take on much of the financial, emotional, and physical responsibilities of caregiving and sustaining households after an AIDS-death occurs within their own household or in households of their kin. Indeed, few differences exist between households with and without a recent AIDS-related death (Madhavan et al., 2009; Schatz, 2007). This may be due to knock-on effects, in which households dealing with AIDS influence other households by requesting assistance and care, including the care of orphaned children after a death, underscoring the need to examine social connections and inter-household connectivity.

A unique feature of South Africa is its strong social grant system. This governmental program provides a non-contributory means-tested pension to all women and men over the age of 60, and it provides smaller but still meaningful grants for children in poor households.^1^ Other social grants—including those for fosterage, orphans, and disability—require additional documentation and thus are less accessible (Booysen and van der Berg, 2005). For rural households with insecure and irregular remittances from working adults, these grants can act as the mainstay, providing food and necessities that sustain the household (Schatz and Ogunmefun, 2007). In the case of older women's pensions, the money can improve the wellbeing of children, particularly girl children living in the household (Case and Menendez, 2007). In rural communities, social grants provide important stable sources of income that open up relational opportunities for credit, respect, and reciprocal dependence.

Most censuses and surveys in Africa use the notion of the “common cooking pot” to delineate shared household membership (van de Walle, 2006). While having practical advantages, anthropologists have been highly critical of the way in which the concept has been used in Africa and elsewhere because it does not reflect important social relationships that extend beyond household boundaries (Guyer, 1981; Hammel and Laslett, 1974). We believe that by expanding the focus of research on female-headed households to include inter-household social connections we will be better situated to identify economic, emotional and physical resources that can mediate hardship. Our analysis gives weight to an emic understanding of the role of financial resources and social connections—including the quality of relationships within and beyond the household—in managing hardship. We explore three main questions: (1) what types of financial resources are most important for stability and maintenance of households under strain? (2) How does a household's place in the larger social environment alleviate or exacerbate hardship in female-headed households? (3) How do financial and social resources buffer female-headed rural households against future hardship?

## 3 Study context

Our study is situated in the Agincourt sub-district in South Africa's rural northeast^2^; formerly part of the Gazankulu homeland, Agincourt is now part of Mpumalanga Province. The Agincourt Unit began conducting an annual household census (births, deaths, and in/out migrations) in the study area in 1992. Most deaths in this area do not occur in hospitals; therefore verbal autopsies are conducted with the next of kin to assess likely cause of death (Kahn et al., 2000). The 2008 census enumerated approximately 84,000 people living in 14,700 households in 25 villages.

In 2007, Mpumalanga had the second highest provincial antenatal HIV-prevalence rate at 32% (http://www.avert.org/safricastats.htm) in the country. In Agincourt, AIDS deaths increased rapidly starting in the 1990s. In 1992, just 1% of all adult deaths were attributable to AIDS but by 2003, that figure had risen to 22% (Madhavan and Schatz, 2007). While in the past women in Agincourt were likely to become heads mainly when their spouses migrated, they are increasingly likely to become heads when spouses or adult children die from AIDS-related causes (Kahn, 2006). As in much of rural South Africa, multi-generational households housing women, their children, and grandchildren are common (Møller, 1998). High rates of unemployment coupled with low levels of subsistence agriculture have resulted in a community largely reliant on migrants' remittances and government sponsored social grants—both of which might be interrupted by an AIDS-related death (Case and Menendez, 2007; Collinson, 2009; Schatz and Ogunmefun, 2007; Twine et al., 2007). Pensions play a particularly important role in this analysis as they offer older female-heads a safety-net not yet available to their younger counterparts. In this sense, the area shares several similarities with rural areas in South Africa, in particular, the effects of labor migration, dependence on social grants, and the rise in female-headed and multi-generational households.

### 3.1 Study description

The Gender and Generation Study, conducted in 2007, used Agincourt census data to generate a stratified random sample of 30 households, in which we conducted semi-structured interviews. A household, in the Agincourt census, is defined as a group of people sharing a common pot of food or resources. However, because of labor migration, it is possible to be counted as a household member even if the person does not physically reside in the house (Collinson, 2009). From each household, we selected two respondents over the age of 17, varying by age and sex, resulting in 58 interviews with individuals between the ages of 17 and 87. The stratified sample included one-third of households with an AIDS-related adult death (as determined by verbal autopsies) in the prior 3 years, one-third with another type of adult death, and one-third with no adult deaths.^3^ Just over two-thirds (21/30) of the sampled households were female-headed, compared to the approximately 40% of female-headed households in the site at large. The oversample of households with an adult death likely contributed to a higher percentage of female-headed households, since wives usually take over headship upon the death of a spouse in this community.

The study focused on gendered and generational roles, responsibilities, and relationships. The semi-structured interviews with all respondents prompted discussions on the same topics: division of labor, patterns of decision-making, intra-household tensions and cooperation, generational contributions to local knowledge, and caregiving strategies for the sick and surviving household members, especially orphans. Study staff conducted and recorded the interviews in the local language, Shangaan, and translated and transcribed them into English.

Our analysis uses the constant comparative method (Glaser, 1965). We read the pair of transcripts from each household against each other; we identified issues and contradictions raised by both respondents and compiled paired summaries. We then read the summaries across households for similarities and differences in the management of hardship, and the ways in which female heads' social environments mediated or exacerbated hardship. We follow the Agincourt definition of “household” in order to extend the findings to the larger population, but we also highlight resource exchange extending beyond households to broader social networks.

### 3.2 Sample characteristics

Our analysis includes households from the AIDS-death stratum, *as well as* households currently or recently providing care to someone sick with AIDS-related illnesses (within or outside of the respondents' household), households affected by the AIDS-death of kin outside the household, and/or households providing care for fostered or orphaned children whose mother and/or father likely have/had AIDS.^4^ By expanding the definition of “AIDS-affected” to include households beyond those with an AIDS-related death, we aim to capture knock-on effects of AIDS (e.g. illness, caregiving for fostered and orphaned children from other households, etc.). Sixteen out of the 21 female-headed households were AIDS-affected—6 that had experienced a death from AIDS and 10 affected in other ways. Table 1 provides the age distribution of all female-headed households and those in the AIDS-death strata; the boldface column represents all female-headed AIDS-affected households included in the analysis.

Most female-heads under the age of 60 (8 of 11, data not shown) became the head upon the death of a husband, who had been the head. Although the majority of older female-heads were widows, as would be expected (6 of 10, data not shown), only three of these heads had recently experienced the death of her husband. More often, deaths in the households of older female-heads were of their son or daughter. These differences stem partly from the age structure of AIDS mortality, in which deaths occur primarily between the ages of 25 and 59. This results in younger female-heads being more likely to have been widowed recently due to AIDS, and in their overrepresentation in the AIDS-death stratum. The fact that households with older female-heads were more likely to be affected by AIDS in other ways brings generational differences to light. Older women are more likely to deal with the illness and death of adult children; younger women are more likely to be affected by the death of spouses.

Older women are affected when their children, who might live outside their household and have young children, become infected with, need care for, or die from an AIDS-related illness; thus, older heads become the locus of care. These ill individuals might be the siblings of the younger heads with whom we spoke. It appears, however, that young heads are less likely than older heads to care for the sick person or for the children of others, and less likely to then take in orphans or contribute their care. Old-age pensions, and to a lesser degree, other social grants, probably play a role here. The stability of income in an older person's house might make it an attractive place to send children.

Table 2 shows household characteristics by the age of household head. Households headed by younger women are on average much larger, and are likely to have nearly twice as many children. Household structure and composition undoubtedly influence the management of hardship, as shown in the analysis of the interview data.

## 4 Female-headed households and their responses to HIV/AIDS

Our analysis examines financial, emotional, and physical wellbeing of the heads and their households in the Agincourt community. Financial security of individuals and households usually is associated with more options to handle stressors. Analyses of financial wellbeing focus on internal *and* external sources of household income, the head's management of household resources, the head's perception of her power and control in difficult circumstances, and her perceived ability to rely on and call on outside sources of support when needed. We use heads' social environment within and beyond households as a lens to examine emotional wellbeing and resources—monetary, physical, and emotional. We emphasize interview discussions related to respect and trustworthiness to highlight how heads interact with and are regarded by their children and grandchildren (their own reports and the reports of the other family member), as well as neighbors and extended family. Our understandings of emotional and physical wellbeing are shaped by the head's health, her feelings about household decision-making, and her ability to access resources in her community.

We identify three points on a continuum of coping—strained/overburdened, essential lifelines/limited stability, and resourceful/stable. These points, assigned based on interview responses, highlight variations in household wellbeing and responses to hardship in this particular rural community. The weakest households include estranged heads, particularly young widows, who are disconnected from social ties and disaffected with their situation. The middle group manages precariously with a limited amount of emotional, physical, and financial support, usually due to one or two external lifelines. In the strongest group, households have a stalwart central figure, as well as strong intra- and inter-household ties. Each continuum point highlights the role of financial and social resources in how female-headed households manage stressors at a given moment. The passage of time and change in access to resources in the household's social environment or in the community at large could shift a household's place on the continuum.

### 4.1 Strained relationships and overburdened

We characterized five heads and households in our sample as estranged and disconnected. They handled substantial burdens without much support. They had fragile social environments with strained relationships within and beyond the household, precarious finances, and experienced loneliness and despondency. Young widows, whose husbands recently died of AIDS, headed three of these households and were likely HIV-positive themselves. These widows had strained relationships with the deceased husband's family regarding funeral decisions, proper mourning, and blame for the death. Unstable financial situations coupled with emotional and physical stress compounded the challenges that death brought to the household as shown in the case of Midah.

Midah,^5^ a 44-year-old AIDS widow, had recently learned of her HIV-positive status and begun treatment. Midah lived with four of her own children (ages 14–23), two orphaned by her husband's sister (ages 12 and 14), and a three year-old grandson. She also continued to assist three orphans who had recently moved out. She reported that even before her husband's death, she had trouble accessing his income for household needs. She long suspected he spent his money on girlfriends. Midah left her husband several times but returned to care for him before he died. After his death, her physical and emotional health deteriorated. She expressed helplessness rather than empowerment in decision-making situations. Midah struggled to secure the respect and discipline of her children and the orphaned nieces and nephews who moved out, something expected by elders in this community. Her pregnant daughter had recently moved in with a boyfriend. Clearly disappointed, Midah told us “I try to show her how life is, but she doesn't want to listen to me.” Her children helped with chores (fetching firewood, cleaning, collecting water from a tap in the yard, etc.), but did so reluctantly. Midah felt ill equipped to handle one son who was misbehaving and doing poorly in school. Her husband's illness and funeral had overwhelmed the household financially. Her husband's employer paid for the coffin, but she reported no other assistance. Her husband's kin did not even visit during his sickness and funeral. Midah had a small sewing business, but she often extended credit to customers who then did not pay. According to her son, “The money she gets doesn't satisfy our needs. If she buys food for the house, the remaining money doesn't buy clothes for us.” Midah's brother occasionally bought shoes for the children, and child grants helped the household, but she had little other support. In short, Midah's need for all types of assistance—financial, emotional, and social—largely was unmet.

Eunice, a 37 year-old widow, similarly was alienated from her three children and husband's kin. Officially, Eunice was the household decision-maker, but in practice her 20-year-old daughter Andiswa assumed responsibility; Andiswa expressed doubts about Eunice's competence. The household struggled financially; Eunice said, “The money we receive from the child grant is not enough to buy food, which will take us for the whole month. In the past the situation was better because my husband bought clothes and food properly.” Despite seeming responsible beyond her years, Andiswa expressed fears, “My father died at a young age and I am panicked for my mother, that she doesn't have a husband. And, I am panicked for my siblings and me. We have only one parent.” Andiswa's one remaining parent had low self-esteem, little social support, and may soon be ill, needing even more care and attention from Andiswa.

Older women widowed long ago, who were caring for orphaned grandchildren at the time of the interviews, headed the remaining two households at this low point on the continuum. Both women were deeply concerned about their ability to carry out newly inherited responsibilities, and about the tone of relationships with their kin. For example, Mercy, age 75, lived with and used her pension to support her son and 20-year-old grandson. The main themes of her interview were unhappiness, ill health, and lack of respect. She worried aloud about her son's promiscuousness, and her grandson, Nhlamulo's failing out of school. Mercy and Nhlamulo both reported their household was poor compared to neighbors and the past. Mercy reported poor health and substantial medical expenses. She was dependent on a daughter, who lived elsewhere, for assistance with transport to obtain medical care, but complained about the lack of reliable help in old age. She expressed feeling disconnected from her son and others who “don't listen to me.” Her son, whose wife left him, had girlfriends coming to the house. Although employed, he was not contributing to the household, exacerbating Mercy's sense of disappointment and abandonment.

Despite the many similarities, these households are not monolithic. For example, relationship strain for younger heads often revolved around young children and the deceased husband's family; for older women, such strain usually came from grown children and grandchildren who showed disrespect or do not contribute sufficiently to the household. However, these households were the most desolate on the continuum because of their overall poor financial, emotional, and physical state, as well as fragile social environments with few or no social connections on which to call in times of need. These female-heads, particularly the older women, saw little hope of change in the future. The younger widows contended that in time they might develop new relationships or find employment to improve their circumstances; however, the likelihood that many of the young widows were already HIV-positive might close even these doors.

### 4.2 Essential lifelines and limited stability

A number of households managed with limited stability. These heads maintained respect and connectedness, but required external assistance. These households generally had sufficient means to feed their families, but most had some social, respect, health, or assistance challenges, issues not uncommon in this rural community. In most cases, just one or two lifelines kept the household from failing financially or emotionally. Additionally, in several cases the deceased's income had helped cover his or her medical expenses or the deceased's burial society covered funeral expenses, thus buffering the impact of AIDS.

One such case is that of Esseny, age 53. Esseny's large household had 13 members and many social connections. Already a great-grandmother, Esseny's son in Johannesburg sent regular substantial remittances. Esseny shared household decision-making power with this son, but made day-to-day decisions in his absence. She worked several odd jobs, including stocking items at a nearby shop with one of her daughters. Of her large household, she said, “I am the mother of all those children, and I am also the grandmother to others. My responsibility is to look after them as a parent. I'm working for them to get food. I'm sewing clothes to sell, and I'm also selling fruit.” Four child grants supplemented the household income. Esseny reported that she took out a loan to start her sewing business and accessed credit at stores when she lacked money to buy necessities. She appeared to have good family and neighborly support, and drew on these in times of need. Rather than being discouraged by her large number of dependents, Esseny saw children and grandchildren as current and future support. “No, the household is not doing well financially,” she said. “Everyone wants money in this household, and everyone is using their own. My neighbors are my brothers. When I look at my situation, I can't compare with them, because I have children and grandchildren. Some of the families do not have children. That means that I've got more support, they do [not] progress because they don't have much support.” Although the household struggled financially, each member contributed labor and assistance to the household. Thus, Esseny viewed her broader social environment as benefitting her and her household as a whole. Her granddaughter mentioned, though Esseny did not, that in 2001 Esseny cared for an adult daughter with tuberculosis, possibly an early symptom of AIDS, suggesting that financial and emotional difficulties may loom in the future.

The case of Octah's small household provides an interesting comparison. Octah was 65, and lived with just two fostered children; her sister was sick, so could not care for them. Octah helped the children and they reciprocated. AIDS affected her house through these fostered children, and a daughter-in-law who died after moving out. Her son and his family, and several other kin moved out over the past year, some still lived nearby. Octah seemed respected, but depressed, lonely and disconnected after her family moved out. She mentioned two recent positive events: “I am happy because I am staying with the children of my sister and they are helping me to fetch water.” And, “My grandson bought bricks and gave them to me and said that he would build a house if he gets more money.” This clear connection between social environment, proximity to kin, and physical place is an important one in this community. Perhaps her “small family” made household finances more manageable. She received a pension, remittances from a son, and help from a grandson, but other children no longer contributed because they were unemployed or had their own households. She had enough extra money this year to buy a cell phone, and generally seemed to make ends meet. However, she reported not doing well compared to neighbors, largely because she felt less connected to kin.

Some female-heads in the middle of the continuum had good support from family and neighbors, as well as pensions and/or a large number of child grants or remittances. Other households struggled financially, but had strong social environments stabilized through family relationships and outside support. Even so, these households did not see themselves as secure but rather as teetering on the edge.

### 4.3 Resourceful and stable

A few heads and their households seemed to manage fairly well despite the hardships their households faced. They had some complaints and problems, but used resources and coped with crises in ways that separated them from the rest. These households were more likely to be headed by older women, but their similarities were otherwise difficult to classify. Each household appeared to have a strong woman who took care of the family, though not necessarily the head. One household exemplifies the complex nature of this theme; this household was managing well, but the head herself was essentially a figurehead.

Dumazile, aged 69, lived with seven people, including two orphaned grandchildren from a daughter who died in 2000. Judith, Dumazile's 35-year-old daughter, revealed that one of Dumazile's sons, who was living in the household, was HIV-positive. Judith, a teacher, assisted with her brother's medical needs, and helped keep his status secret from Dumazile, possibly to protect her. Judith eloquently explained how AIDS was affecting her and their household, “It affected my life because I am always worried that I don't know how must I live with an HIV-person. I don't know how I can tell my children about this. And if I tell them, how will they react toward my brother because they are responsible for washing and cooking for him.” About financial burdens, she said, “I spend a lot of money paying doctors for [my brother] in order to get better treatment. I even consulted without money. When I receive money I then pay the doctor. We made an arrangement that I must come with him even if I don't have the money, and I will pay him back. The problem was with the transport. I have to pay when I go to the doctor because he was unable to walk. Every time I have to pay R150.00 for transport.” Access to health services remains a major obstacle to health care in this rural community. And regarding the emotional costs, she reported, “The difficult part was when giving him a bath because he is a man. My mother used to roll him with clothes on his chest, then wash his back and legs and leave him to finish where she had rolled him. If he was a lady, it would be easy to wash him.”

Although Dumazile was the official head of the household, her daughter was the primary decision-maker. The household depended on Judith's salary, plus four child grants and Dumazile's private pension from her work as a cleaner at a public health clinic. The household was not wealthy, but Judith claimed that they were doing better than in the past and better than neighbors because of multiple income sources and always having food. Still, the son's illness was producing high medical expenses, and Dumazile needed care for high blood pressure and arthritis resulting in visits to various local and more distant doctors, clinics, hospitals, and pharmacies. Despite the costs, this household prioritized seeking health care. To reduce costs, they requested help with transport and expenses from Judith's colleagues and their neighbors when necessary. Although this household appeared to manage well through hardships, the future is uncertain. Rising medical costs for Dumazile's son and related burdens likely will increase financial and emotional adversity. These changes could shift this household downward on the continuum. If Judith was to lose her job or leave the household, the situation could shift even further toward despair.

Dumazile relied heavily on her daughter's support, but in another household, the emotional rock of the family was the older female-head, Thandi, age 66. Thandi lived with three of her sons, three grandchildren, and four great-grandchildren. Her granddaughter Pretty held her in great esteem, “[After my mother died] I felt alone in a strange place. I felt that heaven was over me, I thank God because my grandmother always stayed with me, [so I could] share my ideas with her. Everything I do, I tell her. I can't do anything without her. She is my friend. She is all to us.” This attitude is a reflection of Thandi's respected place in her social environment and offers a partial explanation of why some households fare better than others against the challenges of AIDS in their households.

## 5 Discussion

Our interviews reveal substantial heterogeneity in the coping abilities of female heads and their households in terms of access to financial, emotional, and physical support. Social and geographic isolation created by apartheid have made inter-household social connections particularly important to household survival in rural South Africa. Struggling households have weak social connections and more difficulty coping with AIDS-related disruptions. Female-heads' financial and social wellbeing is particularly important, regardless of age. Interpersonal relationships, social support, social grants, and formal labor opportunities bolster female-heads' wellbeing, which in turn mediates the impact of AIDS-related stressors on their households. Those that are disconnected or lack support from important relatives, such as in-laws or children, are challenged more by AIDS-related disruptions. Such households, lacking familial support, rely heavily on government sponsored social support such as child grants and pensions, something not available in most AIDS endemic areas. Although social grants provide important income for many female-headed households, they are often insufficient to cover even the basic needs, especially for younger heads who cannot access pensions. For younger heads, employment is crucial for household wellbeing, yet is limited in this rural area.

Clear differences exist in the ways AIDS illness and death impact households headed by younger and older heads. Differences often relate to the head's relationship to the deceased (spouse or child), and the place and timing of the illness and death (inside/outside the household and very recent/more distant past). Among households with older female-heads, only one seemed particularly estranged or disaffected, whereas the majority stayed afloat; a few seemed to manage relatively well compared to all the other households/heads in the sample. Our interviews with younger heads and other household members suggest that deaths of husbands not only reduced the number of income-earners, but also created tension with in-laws. Lack of support from a husband's family fractured a young female-heads' social environment, further diminishing her ability to absorb the financial shock of an AIDS-related death. Being younger, these women may not have had the time to build up the social connections beyond husbands' kin, which may be key to absorbing AIDS-related shocks.

Keeping the unique features of the rural environment in mind, policy makers should attend to these generational differences. In our sample, a greater proportion of younger household heads struggled to cope with a recent AIDS-related death, particularly of a spouse, which appears to be a greater burden than other types of AIDS-related impacts. These younger widows appear to have fewer resources for coping with current and future AIDS-related disruptions. Hence, they are more vulnerable and in need of additional assistance. Although many of these households receive social support through child grants, younger heads often struggle to find money for basic household needs. Many undertook informal work to supplement household income, but most seemed overwhelmed by household demands. Such households would benefit from additional grants to younger widows, namely programs that help replace social networks lost to stigma, and employment opportunities for women. These interventions need not continue indefinitely. Time-limited programs may alleviate many immediate stresses related to an AIDS-death. Although income lost to death is permanent, many death-related costs, such as health care debts, funeral, and burial costs, are incurred only once.

AIDS-widows, here shown to be primarily younger female-heads, may be HIV-positive themselves, which may lead to additional problems for their households in the longer term. It is important to note that antiretroviral therapy (ART) rollout in the area may mediate these impacts if individuals are tested and receive treatment. Future research should focus on the likelihood of testing and treatment after the death of a spouse, the wellbeing of children in these households, and the long-term costs and consequences of AIDS. Households headed by older women may also see a shift in the need for resources and social connections with ART rollout in terms of more prolonged costs related to managing illness, but possibly a decrease in young orphaned children in the household. However distance to health care services may continue to be an obstacle. Greater public transportation infrastructure would benefit both young female heads seeking testing, treatment for HIV and AIDS-related illnesses, and older female heads dealing with other chronic age-related health problems.

A limitation of this study is the portrayal of each household at one point in time. The data suggest ways that the proximity of a death both in time and place, as well as the head's age might influence social environments and coping. However, social connections, as well as coping strategies and capacity are dynamic, so the experiences of these households and female-heads are likely to change as events and relationships evolve. Future studies would benefit from tracking households' social connections, coping capacity, and strategies over time in relation to the timing and magnitude of disruptions, such as AIDS-related illnesses, deaths, and caregiving for orphaned children. Tracing rural/urban social and financial connections is an essential extension of this. While similar to the rest of South or Southern Africa, the rural experience in Agincourt may not be comparable to other African rural contexts. For example, connections made through labor migration offer a resource for women, which may not be found in other contexts. Therefore, similar studies should be undertaken in other rural contexts in Africa that are facing HIV/AIDS-related challenges.

## Figures and Tables

**Table 1:** Female-headed households by the age of head.

Age of head	All female-headed households (FHH)	Female-headed households in AIDS-death strata	All AIDS-affected female-headed households

<60	11	5	**7**
60+	10	1	**9**
Total	21	6	**16**

**Table 2:** Characteristics of AIDS-affected households by the age of female-head.

	Households headed by women <60	Households headed by women 60+
Mean age of head	46.9 (34–59)	72.8 (63–87)
Mean household size	8 (3–13)	4.8 (1–11)
Mean number children in household	4.1 (2–5)	1.9 (0–4)
Total number of households	7	9

## Footnotes

1 Historically, age-eligibility for the South African pension began at age 60 for women and 65 for men; beginning in 2008 the age for men was decreased to 63, and then in 2010 to 60 to match women's age-eligibility. Child grant eligibility also has expanded—from eligibility to those under age 8 in 2000 to under age 15 in 2010.

2 The South African Medical Research Council/University of Witwatersrand Rural Public Health and Health Transitions Research Unit (Agincourt) maintains the study site.

3 Of the original 30 randomly selected households, 22 were successfully included in the study. The 8 non-responses included two refusals, one with a recent death, and five households with no one at home. A household in the same stratum was selected as replacement so that the total households remained at 30.

4 Even with substantial data on households from a variety of sources—household rosters, verbal autopsies, and interviews with two household members—it was still challenging to state definitively which households were “AIDS-affected.”

5 All names are pseudonyms.
